# Reversible Myocarditis after Black Widow Spider Envenomation

**DOI:** 10.1155/2012/794540

**Published:** 2012-01-26

**Authors:** Tarek Dendane, Khalid Abidi, Naoufel Madani, Asmae Benthami, Fatima-Zohra Gueddari, Redoune Abouqal, Amine-Ali Zeggwagh

**Affiliations:** ^1^Medical Intensive Care Unit, Ibn Sina University Hospital, 10000 Rabat, Morocco; ^2^Faculty of Medicine and Pharmacy, University of Mohamed V Souissi, 10000 Rabat, Morocco; ^3^Department of Cardiology, Ibn Sina University Hospital, 10000 Rabat, Morocco; ^4^Department of Radiology, Ibn Sina University Hospital, 10000 Rabat, Morocco

## Abstract

Black widow spiders can cause variable clinical scenarios from local damage to very serious conditions including death. Acute myocardial damage is rarely observed and its prognostic significance is not known. We report a rare case of a 35-year-old man who developed an acute myocarditis with cardiogenic pulmonary edema requiring mechanical ventilation caused by black widow spider's envenomation. The patient was previously healthy. The clinical course was associated with systemic and cardiovascular complaints. His electrocardiogram revealed ST-segment elevation with T-wave amplitude. The plasma concentrations of cardiac enzymes were elevated. His first echocardiography showed hypokinesis of the left ventricle (left ventricle ejection fraction 48%). Magnetic resonance imaging showed also focal myocardial injury of the LV. There was progressive improvement in cardiac traces, biochemical and echocardiographical values (second left ventricle ejection fraction increased to 50%). Myocardial involvement after a spider bite is rare and can cause death. The exact mechanism of this myocarditis is unknown. We report a rare case of acute myocarditis with cardiogenic pulmonary edema requiring mechanical ventilation caused by black widow spider's envenomation. We objectively documented progressive clinical and electrical improvement.

## 1. Introduction

The spiders are generally not aggressive unless confined or disturbed. They typically bite on the extremities or when the spider is accidentally squeezed against the body [1]. Black widow spiders (BWSs) are extremely poisonous arachnids, identified by the colored, hourglass-shaped mark on their abdomens. They are found in temperate regions all around the world. In humans, their bites produce severe muscle pain and cramps which may develop within the first two hours. Severe cramps are usually first felt in the back, shoulders, abdomen, and thighs. Other symptoms include weakness, sweating, headache, anxiety, itching, vomiting, difficult breathing, and increased blood pressure.

The black widow spider's venom can cause variable clinical scenarios from local damage to very serious conditions including death. However, acute myocardial damage is uncommon, and cardiac manifestations after a BWS are rarely observed [2–7]. We report a rare case of myocarditis with cardiogenic pulmonary edema (CPE) requiring mechanical ventilation after BWS envenomation. 

## 2. Case Report

**Figure 1:**
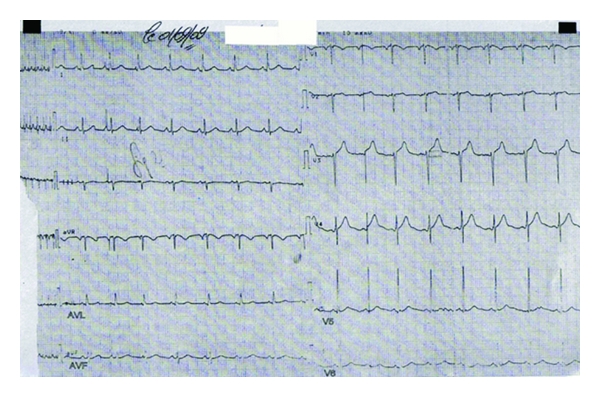
Sinus tachycardia, increase of T wave amplitude in leads V_3_ and V_4_ with 3 mm subsegment elevation ST.

**Figure 2:**
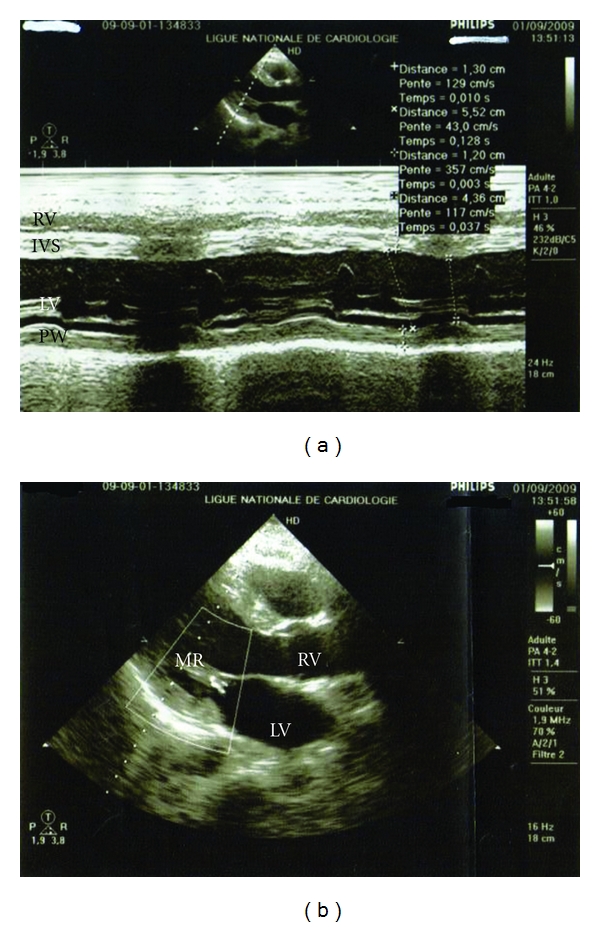
(a) Echocardiographically parasternal long axis depicted improvement of ventricle function. First echocardiogram obtained four days after admission showed LVEF of 48% (calculated by Simpson method). Hypokinesis of the septum and parietal wall of the left ventricle. (b) Second echocardiogram taken 24 days after BWS envenomation depicted good evolution of mitral regurgitation becomes grade I. LVEF was 50%.

A healthy 35-year-old man, single, with no previous cardiovascular history was bitten by a spider while he was walking in Maamoura forest in the northwest of Morocco. He suddenly felt multiples bites on his right leg and two hours later, he declared abdominal pains that rapidly spread to the whole body accompanied by myalgia and sweat with no tremor. The patient and his friend who had seen the creature confirmed that it was a black spider with a large rounded abdomen, commonly known as BWS. He was taken to an emergency department nearly three hours after the bite occurred. The patient initial vital signs were blood pressure (BP) of 221/130 mmHg; heart rate (HR) of 120 beats/minute; respiratory rate (RR) of 20 breath/minute; temperature of 37°C. His initial physical examination was reported as normal except some red spots on his right thigh. His first laboratory results were as follows: white blood cell 35600 per microliter; hematocrit 52%; platelets 281000 per microliter; creatinine kinase (CK) 1140 IU/L (*N* = 100 IU/L); serum bicarbonate 20 meq/L (coagulation profile, kidney, and liver functions were normal). The result of admission electrocardiogram (ECG) was normal except sinus tachycardia. Chest radiography was normal. Nefopam was used to stop his pain and 1 mg of nicardipine was given intravenously, after that he was immediately transferred in the intensive care unit (ICU). At the presentation in our department approximately 6 hours after the spider bite, the patient was still complaining of pain and myalgia in his right leg with no rigidity. He was placed on a cardiac monitor. His vital signs were as follows: BP of 150/60 mmHg; HR of 100 beats/min; RR of 26 breath/min; body temperature of 36.8°C. Chest radiography remained normal. Twenty-four hours after ICU admission, the patient presented agitation, polypnea; RR of 40 breath/minute with no chest pain; tachycardia of 120 beats/minute and O_2_ saturation of 74%. Blood pressure was 140/80 mmHg. Auscultation of the lungs reveals fine crackling rales at the bases. The arterial blood showed PaO_2_ of 52 mmHg on 10 L/min of O_2_; PaCO_2_ of 37 mmHg; pH of 7.39; HCO_3_ of 22 mmHg; SaO_2_ of 86%. Chest radiography showed CPE. The second ICU ECG (Figure 1) showed sinus tachycardia, increase of T-wave amplitude in leads V_3_ and V_4_ with 3 mm elevation of the ST segment. Laboratories revealed a white blood cell count 22 G/L; hematocrit = 49.6%; platelets = 223000 per microliter; urea = 9 mmol/L; creatinine = 101.66 *μ*mol/L; serum bicarbonate = 25 meq/L with otherwise normal electrolytes (Na = 139 meq/L; K = 3.4 meq/L). The troponin I was not checked because of the lack of reactants in our hospital that day. Despite diuretics and non invasive ventilation, dyspnea persisted with decrease of pulse oximeter oxygen saturation requiring tracheal intubation. On day 4 of ICU hospitalization, the patient was extubated successfully. In the same day, his first serum concentration of troponin I was 1.93 ng/mL (*N* ≤ 0.01 ng/mL), and initial echocardiography (Figure 2(a)) demonstrated impaired left ventricle (LVEF 48%, calculated by Simpson method), with midventricular septum, anterolateral, and inferior walls hypokinesia. Mitral regurgitation was moderate. 

**Figure 3:**
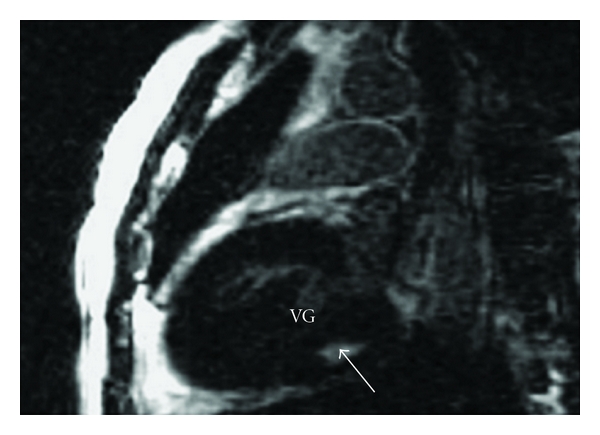
Cardiovascular magnetic resonance T1-weighted sequence obtained two weeks after discharge showed hyperintensity of the posterior lateral wall of the basal left ventricle. LVEF was 57%.

On day 6 of ICU admission, troponin I concentration decreased to 0.027 ng/mL; the ECG recording revealed a normalization of T-wave amplitude in leads V_3_ to V_4_. The patient was put on captopril 25 mg per day and furosemide 10 mg per day, and then he was discharged from the hospital after 8 days of hospitalization. Two weeks after his discharge, echocardiography showed progressive improving LV function of 50% (Figure 2(b)) while the cardiac magnetic resonance imaging (MRI) showed normal-sized ventricles with mild hypokinesia of the LV (LVEF increased from 48% to 57%). T2-weighted imaging confirmed increased signal in the posterolateral wall of the basal LV indicative of hypokinesia (Figure 3).

## 3. Discussion

The BWS is a member of the arthropod phylum and is widely found around the world. Several species of BWS are already found around the Mediterranean [8]. Acute myocarditis can occur after a scorpion sting or a snake bite [9] but it is not a well-known effect of spider envenomation, also myocardial involvement after a spider bite is rare and may cause death [3]. 

The major component of the venom is alpha-latrotoxin, a protein that causes catecholamine release at adrenergic nerve endings and acetylcholine depletion at motor nerve ending. Although the nervous system is the primary target of alpha-latrotoxin, other tissues, such as the heart and lungs, are also susceptible to the toxic effect of alpha-latrotoxin [10, 11]. However, the exact mechanism of myocarditis after BWS envenomation is not known. But in fact, was it an acute myocarditis or Takotsubo myocardiopathy (TM) known as stress cardiomyopathy? In the absence of critical coronary arterial disease, the diagnosis of stress cardiomyopathy should be considered when the history taking reveals that cardiac symptoms were precipitated by intense emotional stress, when there is a unique pattern of left ventricular dysfunction characterized by apical and midventricular contractile abnormalities with sparing of the basal segments, and when there is minimal elevation of cardiac enzymes despite the presence of large regions of focal akinesis in the myocardium [12]. Although many authors [2–6] seem to relate myocardial involvement to direct effects of alpha latrotoxin, we thought initially that there were several findings that should prompt to relate clinical manifestations to the effects of alpha latrotoxin on the autonomic system eliciting massive catecholaminergic release and causing TM. In the present case, the early onset of severe hypertension, followed by the occurrence of myocarditis and pulmonary edema, and the rapid healing of this severe myocarditis in few days and recovery were arguments in favor of TM. Only a few cases of myocardial involvement associated with BWS envenomation have been reported but just three cases with CPE [3, 6, 7]. The different cases are summarized with all features in Table 1. In the present case, the main clinical manifestations of the BWS bite were associated with cardiovascular complaints and precipitated by agitation and emotional stress. ST-T changes in precordial leads have been reported in the study of Pulignano et al. [4]. Erdur et al. reported atrial depolarization abnormalities in postero-inferior territory [5]. The case reported by Sari et al. showed a myocarditis after BWS envenomation with a 0.5 mm ST-segment elevation in leads II, AVF, and V_3_ through V_6_ and accompanying augmentation in T-wave amplitude in leads V_3_ through V_6_ without reciprocal changes [2]. This is similar to the present patient who developed a myocarditis with T wave amplitude in leads V_3_ and V_4_ with 3 mm subsegment elevation ST. However, Pneumatikos et al. reported ECG abnormalities with atrial fibrillation and incomplete right bundle-branch block [3]. ST-T changes in precordial leads were reported in the study of Pulignano et al. The troponin concentration increase and echocardiography abnormalities have been reported in all the studies [2–6]. Pneumatikos et al. [3] reported dilatation of the 4 cardiac chambers and severe global hypokinesis of the LV wall. Pulignano et al. [4] reported akinesis of interventricular septum with depressed left ventricular function. Finally, Erdur et al. [5] reported anteroseptal hypokinesis with depressed LV function recovered within a week. In the present patient, echocardiography found also depressed left ventricular function with progressive improvement over three weeks. The increase in ejection fraction from 48% to 50% does not reflect clinically significant; it is plausible that echocardiography at the time of pulmonary edema would have shown a poorer LVEF. Moreover, this paper is original in having for the first time (in reports on BWS) an MRI examination which is essential to differentiate between acute myocarditis and TM. Thus, in our case T2 weighted imaging confirmed myocarditis with increased signal in the posterolateral wall of the basal LV indicative of hypokinesia, a subepicardial late enhancement with no ballooning LV apex.

## 4. Conclusion

Myocardial involvement after a spider bite is rare and can be fatal. We present a rare case of severe BWS envenomation complicated by myocarditis, CPE requiring mechanical ventilation, and a global, reversible cardiomyopathy. MRI was indispensable to differentiate between myocarditis and TM. We recommend cardiac-specific enzyme with ECG and imagery (echocardiography or MRI) for symptomatic patients and those with underlying cardiovascular disease, bitten by BWS for potential irreversible cardiac damage.

## Figures and Tables

**Table 1:** Cases of spider Bite envenomation with myocarditis involvement.

Age/sex	Bite site	Spider species	Clinical features	Lab findings
15/M	Rt big toe	Black widow spider (BWS)	Local pain; back pain; priapism; abdominal cramps; progressive paresthesia; dyspnea/restlessness; rigors; pulmonary edema	Leukocytosis 18,000; ECG: T-inversion in 1, AVL; S-T elevation: AVL, V_2_, V_3_; highly elevated CK: 2085 IU/L; chest X-ray: pulmonary edema; ECHO: bacmyocardial dysfunction; low ejection fraction 0.264

22/M	Left thigh	BWS (*Latrodectus Hesperus*)	Back and abdominal pain, tremors, diaphoresis, paresthesias, periorbital edema, diffuse muscle fasciculations, pulmonary edema	ECG: incomplete right bundle branch block with ST elevations in the precordial leads, CPK: 243 IU/L, troponin 1c: 1.37 ng/mL ECHO: low LVEF:35–40

16/M		BWS	Typical chest pain	ECG: ST-T changes in precordial leads Echo: akinesia of interventricular septum with depressed left ventricular function

65/M	left foot	BWS	Vomiting, nausea, chest pain.	ECG: 0.5-mm ST-segment elevation in leads II, aVF, and V_3_ through V_6_ and accompanying augmentation in T-wave amplitude in leads V_3_ through V_6_ Troponin: 6.1 ng/mL Echo: normal

22/M	left shoulder	BWS	Anxiety, severe hypertension, nausea, vomiting, tremor, generalized pain,diaphoresis, and rhabdomyolysis	Troponin I: 0.75 ng/mL, ECG: atrial depolarizationabnormalities in leads D II, III, and aVF, and depolarization abnormalities in leads V_1_ and aVL. LVEF: 50%, anterior and septal wall motion abnormality
